# ‘Safe Sport Is Not for Everyone’: Equity-Deserving Athletes’ Perspectives of, Experiences and Recommendations for Safe Sport

**DOI:** 10.3389/fpsyg.2022.832560

**Published:** 2022-03-07

**Authors:** Joseph John Gurgis, Gretchen Kerr, Simon Darnell

**Affiliations:** Faculty of Kinesiology and Physical Education, University of Toronto, Toronto, ON, Canada

**Keywords:** safe sport, equity-deserving, athletes, discrimination, interpretive phenomenological analysis

## Abstract

There is a growing concern that the voices of athletes, and in particular, athletes from equity-deserving groups, are unaccounted for in the development and advancement of Safe Sport initiatives. The lack of consideration of the needs and experiences of diverse groups is concerning, given the existing literature outside the context of sport indicating that equity-deserving individuals experience more violence. As such, the following study sought to understand how equity-deserving athletes interpret and experience Safe Sport. Grounded within an interpretive phenomenological analysis, semi-structured interviews were used to understand how athletes with marginalised identities conceptualise and experience Safe Sport. Seven participants, including two Black male athletes, two White, gay male athletes, one Middle Eastern female athlete, one White, female athlete with a physical disability and one White, non-binary, queer, athlete with a physical disability, were asked to conceptualise and describe their experiences of Safe Sport. The findings revealed these athletes perceived Safe Sport as an unrealistic and unattainable ideal that cannot fully be experienced by those from equity-deserving groups. This interpretation was reinforced by reported experiences of discriminatory comments, discriminatory behaviours and systemic barriers, perpetrated by coaches, teammates, and resulting from structural aspects of sport. The findings draw on the human rights literature to suggest integrating principles of equity, diversity and inclusion are fundamental to safeguarding equity-deserving athletes.

## Introduction

In response to several high-profile cases of athlete abuse (Kennedy and Grainger, 2006; Kelly, 2013; Masters and Veselinovic, 2018), many sport organisations have committed to developing policies and programmes to advance what has come to be known as Safe Sport^1^ (Gurgis and Kerr, 2021). Several philosophical conceptions of Safe Sport have emerged, grounded in interpretations related to abuse prevention in sport. For example, consensus statement of the International Olympic Committee (1997) on harassment and abuse defined Safe Sport as ‘an athletic environment that is respectful, equitable and free from all forms of non-accidental violence to athletes’ (Mountjoy et al., 2016, p. 2). In comparison, the Coaching Association of Canada (2020, para. 1) described Safe Sport as the collective responsibility of all sport stakeholders ‘to create, foster and preserve sport environments that ensure positive, healthy and fulfilling experiences for all individuals’ and to ‘recognize and report acts of maltreatment and prioritize the welfare, safety and rights of every person at all times’. Although the term ‘Safe Sport’ has been used across North America and internationally (e.g., Sport Singapore and Safe Sport Africa), other regions and organisations have used the term ‘safeguarding’ (Hayhurst et al., 2016). For example, the Child Protection in Sport Unit in the United Kingdom (Boocock, 2002), which existed long before other Safe Sport organisations, uses the term safeguarding, defined as:

Protecting children from maltreatment; preventing impairment of children’s mental and physical health or development; ensuring that children grow up in circumstances consistent with the provision of safe and effective care; and taking action to enable all children to have the best outcomes. (Department of Education, 2018, p. 7)

For the purposes of this paper, the term, Safe Sport, will be used.

The proliferation of Safe Sport initiatives stemmed from high-profile cases of athlete abuses as well as various prevalence studies indicating the frequency with which athletes from various levels of sport experience harm (Alexander et al., 2011; Vertommen et al., 2016; Parent and Vaillancourt-Morel, 2020; Willson et al., 2021). In addition to highlighting the prevalence of harmful experiences, these studies suggested that athletes in equity-deserving^2^ groups experience more harm in sport. For example, across numerous studies, researchers have shown that girls and women are more likely to be victims of various forms of abuse. Alexander et al. (2011) conducted a prevalence study to examine children’s experiences of harm in organised sport in the United Kingdom; the findings revealed that female athletes, more so than their male counterparts, reported more frequent experiences of certain types of emotional harm, including being embarrassed or humiliated, bullied, criticised for weight/looks, being threatened and being ignored in ways that makes one feel badly. Additionally, more female than male athletes strongly agreed that their sport experience contributed to poor body image and taught them to dislike their body. Similarly, in a prevalence study conducted by Vertommen et al. (2016), females reported significantly more experiences of sexual violence in sport compared to males. In Canada, Willson et al. (2021) explored the prevalence of maltreatment experienced by current and retired Olympic and Paralympic athletes; both current and retired female athletes reported significantly more experiences of neglect, as well as psychological, physical and sexual harm, than their male counterparts. Additionally, findings from the 2020 Athlete Culture and Climate Survey launched by the United States. Center for SafeSport, which surveyed 3,959 adult athletes across 50 sports, found that women and gender-nonconforming athletes experienced higher rates of psychological harm and neglect than those athletes who identified as men.

Although only a few studies have explored experiences of interpersonal violence or maltreatment among other equity-deserving groups, findings suggest that athletes who identify as lesbian, gay or bisexual report more sexually harmful experiences than their heterosexual peers (Vertommen et al., 2016; Willson et al., 2021). Additionally, prevalence studies have shown that racialized athletes report more experiences of physical harm (Vertommen et al., 2016; U.S. Center for SafeSport, 2021; Willson et al., 2021). More specifically, the U.S. Athlete Culture and Climate Survey found that Black, Asian and Multiracial athletes were more likely to experience physical harm, and Black athletes experienced nearly double the rate of inappropriate sexual contact, as well as greater experiences of sexual assault (U.S. Center for SafeSport, 2021). Regarding athletes with a disability, findings from prevalence studies have been mixed. The data from the United States Climate Survey revealed that athletes with a disability were more likely to experience psychological harm, neglect, and inappropriate sexual contact (U.S. Center for SafeSport, 2021). Similarly, Vertommen et al. (2016) reported that athletes with a disability reported greater experiences of interpersonal violence than those without a disability.

Research has also highlighted the prevalence of racial (Burdsey, 2011) and gender-based (Kaskan and Ho, 2014) microaggressions in sport. Microaggressions refer to frequent experiences and behaviours that reinforce stereotypes, biases, forms of prejudices and oppression (Strunk and Locke, 2019). Although microaggressions are understood to be subtle, the cumulative, consistent and pervasive nature of these experiences can have severe negative effects on the physical and psychological welfare of individuals (Sue et al., 2007). Findings from the United States Athlete Culture and Climate Survey indicated that 72.4% of athletes reportedly experienced anti-inclusive behaviour (i.e., microaggressions), such as exclusion or invalidations, and 48% of athletes reported experiencing discrimination (U.S. Center for SafeSport, 2021). Similarly, Joseph et al. (2021) reported that student-athletes affiliated with Ontario University Athletics experienced microaggressions in the forms of implicit racism, explicit and overt racism, mistreatment and differential treatment, assumptions of athlete wealth and poverty, player (non-) recruitment, lack of support for athletes and additional labour.

Taken together, the existing research on maltreatment of athletes indicates that athletes from equity-deserving groups report more experiences of some harms. To date, these differential experiences have been indicated through quantitative surveys of prevalence. However, the perspectives of Safe Sport from athletes from equity-deserving groups have yet to be explored. Therefore, this study seeks to contribute to our knowledge by exploring the Safe Sport perspectives and experiences of athletes from various equity-deserving groups through a qualitative approach. Specifically, the current study aimed to address the research questions of: (1) What are equity-deserving athletes’ perspectives of Safe Sport? and (2) How do equity-deserving athletes experience Safe Sport? The study’s focus forefronts the voices of equity-deserving athletes and thus, dismantles the ‘top-down, “expert”-driven approach’ (Frisby and Ponic, 2013, p. 393) of Safe Sport that appears to have perpetuated inequalities in sport by disqualifying the voices of equity-deserving athletes. Together, equity-deserving athletes’ experiences of microaggressions, and research indicating increased exposure to maltreatment, suggest that consideration of the idiographic experiences of equity-deserving athletes is needed.

## Methodology

This study is methodologically informed by interpretive phenomenological analysis (IPA). IPA is a methodological strand of phenomenology, developed ‘to allow rigorous exploration of idiographic subjective experiences …and social cognitions’ (Biggerstaff and Thompson, 2008, p. 215). IPA explores in depth the lived experiences of an individual and how they make sense of their personal experiences (Smith, 2004). IPA is classified as a double hermeneutic; as researchers, we attempt to make sense of participant experiences, who are simultaneously attempting to make sense of their own social and personal world (Smith, 2004). Successful IPA research requires the researcher to gather reflective, thorough, original data from the research participants and for the researcher to offer an interpretation of the data that is grounded in the shared accounts or other psychological concepts; these processes are referred to as ‘giving voice’ and ‘making sense’ (Larkin and Thompson, 2012, p. 101). In this study, the phenomenon of interest is Safe Sport and how it is experienced and understood by equity-deserving athletes. This methodology was purposefully selected to make sense of the social conditions that affect equity-deserving athletes’ interpretations and lived experiences of Safe Sport.

### Participants

Interpretive phenomenological analysis studies are often conducted with small and homogenous samples; the quality of data gathered through a small sample size, rather than the quantity of data, is meant to generate insightful analyses (Larkin and Thompson, 2012; Tamminen et al., 2013). The following study sought to understand how current and retired high-performance equity-deserving athletes understood and experienced Safe Sport. Conducting research on equity-deserving participants is meant to enhance our awareness of the various inequalities that manifest ‘from the complex interactions of social identities’ (Simien et al., 2019, p. 410) and enrich our understanding of the oppressive societal and environmental factors that make sport unsafe for underrepresented participants. Further, pursuing Safe Sport research with equity-deserving athletes may inform how to effectively address systemic barriers of inequality that perpetuate unsafe experiences for athletes with marginalised identities. Finally, given that athletes are generally considered the most vulnerable population involved in sport (Dean and Rowan, 2014), it is crucial to understand the experiences of vulnerability endured by equity-deserving athletes, who may be susceptible to further harm because of their identity.

For this study, seven high-performance (e.g., inter-university, Olympic and Paralympic) athletes from Canada were recruited using purposeful and snowball sampling, which permitted us to intentionally recruit participants perceived as possessing specific knowledge or experience related to the topic of interest (Vogt, 2005; Creswell and Plano Clark, 2011). We purposefully sought participants who fit the criteria of the study (i.e., equity-deserving, minimum 18 years and current or retired athlete), using email addresses that were readily available on academic, inter-university and sport organisation websites. Recruitment emails were sent to eleven participants, of which four responded. Following our interviews with the four consenting participants, we asked each athlete if they could recommend another 1–2 athletes who may be willing to participate in the study. We were provided eight recommendations, of which three agreed to participate.

Paul was a retired inter-university basketball player, who had been out of sport for two years and identified as a Black, Muslim and heterosexual man. James was a current inter-university basketball player, who identified as a Black, Christian and heterosexual man. Mary was a retired inter-university swimmer, who had been out of sport for one year and identified as a Middle Eastern, Coptic Christian and heterosexual woman. Ruth is a current Paralympic swimmer who identified as a White, heterosexual woman with a physical disability. Thomas was a retired inter-university swimmer, who had been out of sport for two years and identified as a White, gay man. Peter was a retired Olympic swimmer, who identified as a White, gay man. Finally, Danielle Peers is a retired Paralympic wheelchair basketball player who identified as White, non-binary, queer and as having a physical disability.

Although six of the seven participants agreed to the use of a pseudonym to ensure confidentiality was preserved, Danielle Peers (hereon referred to as Danielle) chose to waive confidentiality in favour of being identified. Consistent with the recommendations advanced by the Tri-Council Policy Statement (Canadian Institutes of Health Research, 2018) on Ethical Conduct for Research, we clarified the potential professional and personal risks of participating in the study without a pseudonym and obtained written consent from Danielle confirming disclosure of their identity. We respected Danielle’s desire to be credited for the contributions to the research and recognised that the disclosure of Danielle’s name would not jeopardise the identity of other participants, which further supported our decision to refrain from assigning a pseudonym (Canadian Institutes of Health Research, 2018).

### Data Collection

An interview guide was prepared and included the following areas of conversation: demographics, background and rapport-building, knowledge of Safe Sport, personal experiences of Safe Sport, facilitators and barriers to experiencing Safe Sport, recommendations for advancing Safe Sport and final thoughts. When used with IPA studies, an interview guide is intended to be flexible to enable participants to describe events they perceive to be meaningful (Biggerstaff and Thompson, 2008; Smith et al., 2009). Participants were asked questions such as: ‘What does Safe Sport mean to you?’, ‘What are your personal experiences of Safe Sport?’, ‘Has your identity influenced your sense of safety in sport? If so, how?’ and ‘How do you think people of various identities (e.g., race, sexuality and disability) experience Safe Sport?’

Individual, semi-structured interviews were conducted face-to-face *via* the video conferencing platform Zoom Video Communications, necessitated by the pandemic-related restrictions that limited in-person interviews. The inclusion of open-ended questions in IPA studies is imperative so that participants feel they have opportunities to describe their personal experiences and understanding of the phenomenon (Jones et al., 2014). The interviews ranged between 60 and 130 min and with the consent of the participants, were audio-recorded and transcribed verbatim. Follow-up interviews were conducted with three participants to clarify and collect information to further our understanding of Safe Sport as a phenomenon (Alase, 2017).

### Data Analysis

Parson (2019, p. 16) indicated ‘considering one’s positionality is the first step towards conducting research that contributes to more equity in society, instead of reproducing inequity or diminishing it’. Positionality requires the researcher to critically examine their social position within various power structures (Strunk and Locke, 2019). Jones et al. (2014) also emphasised the importance of researchers being constantly reflexive when conducting phenomenological research. Patton (2002, p. 485) explained ‘internal reflection allows for a phenomenological attitude shift and makes pre-understanding clear’. Prior to data analysis, the principal investigator (PI) underwent a thorough process of self-reflection, which involved adherence to the reflexive processes of epoché and bracketing (phenomenological reduction; Berry et al., 2010). Using a reflexive journal, the PI attempted to fully acknowledge and set aside normalised interpretations of Safe Sport as a demonstration of his commitment to openly exploring the experiences and understandings of Safe Sport held by equity-deserving athletes (Moustakas, 1994). The pre-determined understandings the PI set aside before conducting interviews revolve around *a priori* knowledge he possesses within the area of Safe Sport, gained through the privileged access of various resources made available to him as a researcher. It was critical for him to be self-aware that others may not share the same view or knowledge pertaining to Safe Sport, nor may they have access to the resources that illuminate one’s awareness and understanding of these concepts. Additionally, the PI engaged in ongoing conversations with the research team who were perceived as critical friends (Sparkes and Smith, 2013); this process aided the PI with clarifying personal beliefs, confronting assumptions and exposing pre-conceived understandings related to the Safe Sport experiences of equity-deserving athletes (van Manen, 1990). As a heterosexual, able-bodied and Christian male who identified as a person of colour, it was critical that the PI did not project personal beliefs or biases regarding the various conditions affecting Safe Sport, including identity. By not projecting personal views of Safe Sport on the participants, the PI allowed for organic, untampered experiences of Safe Sport to be introduced and discussed as experienced or understood by equity-deserving athletes.

The data analysis followed six steps of Sparkes and Smith (2013, p. 128) to conducting an IPA. Initially, the transcripts are read and re-read multiple times to develop a sense of understanding of the participants’ stories. During this process, we recorded notes and personal thoughts regarding the events depicted within the transcripts. Then, we began to identify and label themes. At this stage, the analysis progressed to a higher level of interpretation focused on “finding expressions which are high level enough to allow theoretical connections within and across cases but which are still grounded in the particularity of the specific thing said” (Smith and Osborn 2008, p. 68). For example, throughout this step, we observed that several athletes shared experiences of discrimination in sport, which they perceived as contributing to an unsafe environment. The initial themes were then connected by looking for higher-order relationships between one another. For example, we found some experiences of discrimination occurred verbally, while other experiences occurred non-verbally, through acts of exclusion. Then, a table was used to elaborate on our themes and establish connections, thus providing a clearer understanding of Safe Sport. We continued our analysis by searching for patterns among other cases, thus attempting to identify similar patterns among emerging themes, with a commitment to identifying new issues that emerged within the transcripts. For example, in this step, we identified patterns suggesting Safe Sport to be an unrealistic and unattainable ideal for equity-deserving athletes.

## Results

### Equity-Deserving Athletes’ Perspectives of Safe Sport

For many athletes, there was uncertainty about the meaning of Safe Sport. Participants interrogated current interpretations of Safe Sport and raised doubts about whether Safe Sport was relevant, realistic or attainable for equity-deserving athletes. For example, Thomas (White, gay man) admitted:

It [Safe Sport] does not seem real, at least not for me. I’m gay and so Safe Sport seems idealistic. In theory, sure Safe Sport is happy and fun and accepting and all that good stuff. I did not experience that, probably because I’m gay. So Safe Sport seems like an unrealistic load of crap.

Paul (Black, heterosexual man) echoed similar sentiments, as he suggested that Black athletes are denied the benefits of Safe Sport that White athletes appear to often benefit from:

As of right now I don’t think a Black person can experience Safe Sport like a White person does. Unless they’re willing to address the whole race issue. If they’re not able to speak up, see eye to eye, and recognize these Black athletes as more than people they are using for money, but people they respect too, that’s when Black people will be able to have a better definition for themselves of Safe Sport.

Additionally, Paul (Black, heterosexual man) expressed his concerns of Safe Sport needing to be earned by Black people, and others who identify as equity-deserving:

I feel like I have to prove myself as a human or as a person because I’m at a disadvantage for being Black… it’s almost like I need to earn Safe Sport because I’m Black. So once people accept me for my skin colour, then I can feel safe in sport…it’s probably the same for Brown people, people in wheelchairs, or gay people too. We’re not easily accepted by others so we need to prove ourselves and once we do, then we can benefit.

Similarly, James (Black, heterosexual man) agreed that Safe Sport was an ideal that could not be fully experienced by Black athletes:

It’s up in the air if Black athletes can ever receive Safe Sport. There are a lot of uncontrollables in Safe Sport… It’s hard being a Black athlete…it’s not as safe as being a White athlete. You’re not favoured…Black people or people of colour won’t experience Safe Sport in certain instances…there are people that are White that are in power, coaching staff, head of athletics…you need to be better than your White counterparts to succeed. That adds to the pressure of being a Black athlete in a White majority. Athletes of colour can experience Safe Sport to an extent, but not fully.

Again, we see how contemporary conceptualizations of Safe Sport do not accurately reflect the safety and wellbeing of all athletes equally, with Black and gay athletes being at an apparent greater disadvantage. Additionally, Ruth (White, heterosexual and physically disabled woman) suggested that Safe Sport looks different for participants with varying abilities:

I think Safe Sport is different for everybody because everybody has different needs. I think the definition can stay the same in general terms; there’s core values that should apply to everyone. Not feeling discriminated against, feeling safe alone in a room with a coach or an official or somebody that’s higher up in authority, making sport fair and equal for everyone, making sure people feel healthy and happy, but you have to consider more factors for Para-athletes. Para-athletes have a range of abilities; some are strictly physical or cognitive and some athletes have both. What Safe Sport means to those individual athletes will differ because they experience safety differently in response to their unique challenges.

Ruth’s broad interpretation of Safe Sport embraces principles that apply to everyone alike; however, she recognised how the distinct characteristics that distinguish athletes from equity-deserving groups from societal-normative groups (i.e., White, male, heterosexual and able-bodied) renders safety a unique experience for different athletes. In defining Safe Sport, Danielle, a White, non-binary, queer and Paralympic athlete, raised questions of legitimacy:

Safe Sport to me is about seeing how certain harms are unequally distributed and how the benefits of sport and opportunities to play sport are unequally distributed. Who gets to bring their whole self to sport and who gets to bring a small fraction of themselves to sport to be safe? What kinds of contortions do people have to do to be okay when they are playing sport? I would be on the spectrum of the broadest possible definition as to what degree does sport perpetuate harm or sporting environments perpetuate harm, produce harm, larger social harms, refuse to insulate people from or resist social harms that are largely socially available. To what point do we refuse harm reduction and refuse harm recognition as parts of Safe Sport? So, very broad.

The questions posed by Danielle further supported the claims made by the other athletes that Safe Sport is advantageous to normative participants of sport.

Although the relevance of the concept of Safe Sport to the participants was questioned, Safe Sport was interpreted as extending beyond physical safety to the advancement of positive values, such as inclusion, fairness and acceptance. For example, Paul (Black, heterosexual man) described Safe Sport as:

Any activity or sport that has no boundaries in terms of who you are. You should not feel unsafe or uncomfortable because you are gay, a Christian, Black, or Asian. And I know people do feel that…I’m Black, so no oppression, no biases, in terms of facing the other teams or people…It’s important that safe does not just mean ‘I’m not going to get injured’, but it also refers to a fair playing field. I’m not going to be nervous that my opponent may injure me or that he’ll have the upper hand because I’m Black and he’s White or because the ref. is White…Your identity should not interfere with your right to play, but it does and so we will not have Safe Sport until that changes.

Paul’s definition of Safe Sport goes beyond interpretations of physical safety to acknowledge the importance of fair play and self-expression in sport. Similarly for Peter, a White, gay man, Safe Sport was understood as an environment that encouraged expression and penalised those who silenced and oppressed others:

I somehow picture an environment where people feel comfortable in their own skin, if something inappropriate happens, there’s recourse, process, some mechanism that says this is not okay…The safeness starts when people feel they can speak out to defend someone that is being abused…A lot of sport administrators from my niche in the LGBTQ[2S] world will not come out because all sorts of horrible things get thrown and confused and there are certain people on the other side of society that think if you are working with kids and you are gay that you are a paedophile. There are some really scary things that get promoted out there for coaches.

### Equity-Deserving Athletes’ Experiences of (Un)Safe Sport

#### Discriminatory Comments

When asked about experiences of Safe Sport, participants disclosed experiences of discrimination surrounding race, ability, sexuality and/or gender. The use of verbal microaggressions, characterised by insensitive or derogatory comments, was one way in which athletes experienced discrimination. Ruth (White, heterosexual and physically disabled woman) shared personal experiences of bullying from teammates:

I hear what others say. ‘Why is she even here?’ ‘Go easy on her.’ ‘They feel bad for her.’ ‘She should swim with her kind.’ It’s hurtful. I struggle because I see myself as an athlete. You ask me, I’m a swimmer. I’m not a female swimmer, I’m not a disabled swimmer, I’m a swimmer.

Peter (White, gay man) was also exposed to an unsafe sport environment, characterized by frequent use of homophobic language:

I’ve had the word ‘fag’ follow me around for a long time. In school that was the case. I made the mistake of hitting on an Australian guy who I thought was gay when I was about 19 at a swim meet, and all the Australian team thought I was a fag. The vernacular in the locker room lends itself to that as well. That’s where much of our hazing took place. I’d rather not get into that. It’s there. I’d rather not dig it up. It was not pleasant, and it was always around ‘fag’ and ‘get down on your knees’, that sort of stuff.

Similarly, Thomas (White, gay man), a retired inter-university swimmer, admitted to being addressed negatively because of his sexuality. Thomas expressed ‘It’s almost as if they saw being gay as my weakness. As if it made me less of a man…I’d be called “pussy,” “fairy.”’

Verbal microaggressions in the form of stereotyping were commonly reported by each Black athlete. Paul (Black, heterosexual man), a retired inter-university basketball player, shared:

I had teammates, Black guys and non-Black guys, who would tell me to start playing like a nigga. Not nigger, nigga. For them, nigger was a proper Black man, whereas nigga was a tougher version of a Black man. A nigga knows how to ball, is fast, flashy, and doesn’t take shit from anyone…it was their way of telling me to toughen up and play aggressive…obviously there are better ways to motivate someone. I didn’t personally like the label…It [racial slur] brings us [Black people] down.

Similarly, James (Black, heterosexual man), who was currently competing as an inter-university basketball player, reported experiences of stereotyping perpetrated by his coach:

I’ve experienced microaggressions from coaches on my team. They would say questionable things at the time like, ‘you can jump high or run fast because you’re Black.’ He did it in a way not to harm me, but to encourage or support me or make me laugh. There was a big misconception of his understanding of microaggressions and how that can negatively impact someone. It’s toxic. Doing that over the 4 years…those things weren’t cool. Not just to me, but to other players of different cultures. Even White people, he would tell them they can’t jump because they’re White.

In addition to experiencing racial microaggressions, James (Black, heterosexual man) admitted that his coach would frequently make insensitive comments based upon faith:

I know me and some of the Christians on the team would get heat at times and made fun of by certain coaches. I remember my first year, we were trying to get pre-game prayer as one of the things we would do before the game and our coach was hesitant to put it in there and he’s wondering why we would even suggest it. There were a lot of side jokes about Christianity by coaches. Like, ‘you guys are with Athletes in Action but you’re athletes that get no action.’

Finally, Mary (Middle Eastern, heterosexual woman), a retired swimmer, described how training alongside male athletes made her susceptible to various misogynistic stereotypes:

Just stupid comments, like ‘should not you be in the kitchen?’ Or ‘is it that time of month again?’…other girls got it just as bad. I think it comes with the territory of training with guys. In swimming we do everything together, whether its practice, lifts, or tournaments. So, you get caught up in the crossfire of what people call locker room talk…it does not help that we tend to be sexualized a lot more because of what we wear, so from time to time you catch guys staring or catcalling. They have no right, but that does not stop them.

As a Middle Eastern woman, Mary noted how some teammates questioned the appropriateness of her competing in sport:

The comments [teammates] made about my ethnicity would go overboard at times. Guys asked if I received permission from my father to swim and whether I was going to get stoned for showing my skin and hair…There was this one time when a guy threw a towel on my head and called it the swimmer’s hijab, which was just disturbing. And I’m a Christian Middle Eastern, so we aren’t required to wear a hijab…just ignorant and disrespectful.

For Mary (Middle Eastern, heterosexual woman), the intersections of her ethnicity, gender and faith exposed her to verbal microaggressions perpetrated by teammates who held stereotypes related to being a Middle Eastern Christian woman.

#### Discriminatory Behaviours

Many participants reported experiencing behavioural microaggressions based upon how they identify and are presumably perceived by others. As Thomas (White, gay man) described:

I had a teammate who always thought it was funny to pair me with the girls whenever we had group training. Guys would put tampons in my gym bag or order me girly drinks at team functions…you know the saying, ‘one of the guys?’ To some of them, I was one of the girls.

Danielle (White, physically disabled and non-binary queer) elaborated on the coercive and insensitive tactics used by able-bodied coaches against athletes with physical disabilities:

I’ve had coaches that sent memes to athletes about three-legged dogs and paralysed dogs to inspire us to play better in the next game. Non-disabled folks sending these things along…even within our own community. I think of the internal violence that disabled athletes do to each other in terms of the mocking of Special Olympics by Paralympians. The devaluation of the competition by particular athletes…The devaluation of disabled athletes, athletic capacities, experiences, the way systemically disabled people are blocked from becoming leaders in their own sports…I’ve experienced and seen in athletes an incredible amount of harm to do with the amount of continual surveillance and coercion from able-bodied coaches…I’d say having an impairment that isn’t stable and static made me more subject to scrutiny around classification and certainly made and set me up to be forced or coerced to do things by my coaching staff that were explicitly against the agreed upon protocol that would keep me safe.

For Danielle (White, physically disabled and non-binary queer), the internet became an avenue for different able-bodied stakeholders to disrespect and criticise those with a physical disability. Additionally, Parasport, characterised as a space of surveillance and coercion that is often dominated by able-bodied coaches, seemingly created a culture of sport perceived as further suppressing athletes with a disability.

A few participants described feelings of being excluded from sport because of how they are perceived by others, as illustrated by Paul (Black, heterosexual man):

My coach wouldn’t play me and there was never an explanation why. Before I made the team, there was another Black player who I knew from club, who was playing and eventually quit. He found out I was playing and told me, ‘don’t expect much playing time; coach doesn’t play Black people.’ That’s literally what he said. I was shocked. I definitely felt a prejudice feeling toward Black players. That whole situation that I’ve been through opened my eyes to …the racial issues in sport. It made me think, what else is going on and what are they thinking when I’m in practice or when they’re making the starting lineup for the game.

Paul explained how he eventually quit the team due to a lack of playing time. Paul’s experience portrayed how perceived discriminatory coaching practices can negatively impact the sport experience, to the extent that athletes question their participation or withdraw from sport.

#### Systemic Barriers

Although not as commonly addressed, some participants introduced how high-performance sport, or more broadly, the physical and socio-cultural organisation of sport spaces, may at times function to exclude certain groups. Ruth (White, heterosexual and physically disabled woman) lamented about the ableist mentality of inter-university sport, which she believed prevented her from competing for her university, despite being a Paralympic swimmer:

I was good enough to compete in Rio, but I’m not good enough to compete for my university. I know that’s not true, but [university] doesn’t allow me to compete in [national] competitions. It’s as if I’m getting punished for being disabled. My right to compete is taken away from me because my able-bodied opponents have an apparent advantage, even though I set standard…times in practice. It’s a very ableist culture. [University] wants to show they’re forward thinking by taking someone like me on the team, but it’s just able-bodied people telling disabled people what they can or can’t do with their body.

Danielle (White, physically disabled and non-binary queer) echoed similar frustrations about the inaccessibility of certain spaces in and out of sport:

90% of the places I go I cannot enter…being non-binary and queer was a thing I could never bring into sport. I cannot be involved in any of the Gay Games or Out Games because there’s no place for someone of my embodiment to exist in those sporting cultures…there is an entire realm of sport I cannot access even in a wheelchair. I do not even get to experience the microinvalidations because I cannot even enter the room…It’s not about the accommodations or support, it’s that people actively continue to build these spaces…it’s not about me not having accommodations, it’s that society continues to perpetuate a society in which only some people get to participate in that society.

The exclusion of certain participants based upon gender, sexuality or ability may be a consequence of the binary categories embedded in the organisation of sport that subsequently disqualify individuals who do not fall within said categories. Peter (White, gay man) explained:

Sport tends to make those things so binary. If you’re not masculine, you must be feminine and if you’re not feminine, you must be masculine…it’s a harmful setup because it naturally excludes those who feel they don’t fit the criteria to participate in one or the other and it doesn’t consider the unique differences of those who fall outside mainstream classifications of sport.

Finally, Paul (Black, heterosexual man) explained how the primarily White leadership of sport reproduces conceptions of safety that overlook discrimination:

Safe Sport is really White…I think Black people could feel a little safer in sports when there are Black leaders making decisions. I think every sport has a leader that’s White…You have White people making rules for people who don’t identify as White. How would a White person understand my struggle as a Black man? …White people don’t experience discrimination the way [Black people] do…it can be scary for us. When you’re targeted because of your skin colour, it’s just unsettling.

Overall, the Black athletes in particular raised questions about the relevance of Safe Sport and abilities to experience it when White leaders are establishing Safe Sport parameters.

#### Recommendations for Advancing Safe Sport

Many participants offered recommendations related to advocacy, education and community engagement to advance a culture of Safe Sport characterised by principles of equity and inclusion, rather than abuse prevention. For example, after being targeted by a racial slur, James (Black, heterosexual man) used his social media presence to advocate for better treatment of Black athletes:

I stood in front of my car and cried after the game. ‘NIGGER’, in big letters, keyed right across my door. No doubt I was targeted; the coward that did it knew it was my car…I was so angry and my AD [athletic director] who’s White told me a week after, ‘I’m sorry about your car.’ That’s it…I decided to post about it. I have a large following and know I’m a role model to others, so I felt that I had to bring this to everyone’s attention. I wasn’t going to ‘shut up and dribble’, it’s been too long of this shit for Black athletes.

When asked to describe the benefits of posting his experience on social media, James (Black, heterosexual man) continued:

There’s no diversity in my school, not even among the leaders, so it never seems like people are listening when I bring things up. They just don’t understand. But people listen to me on social media, so I use it because it’s my way of protesting against the shit I have to put up with. Kaepernick knelt for what he believed in and before that there was [Tommie] Smith. I want to be a good role model to others. I want to show people I’m not violent, that I’m not a thug. I’m a Christian basketball player in university and I happen to be Black. I shouldn’t have to struggle any more than the next guy… I think it did some good. People were talking, people were reposting, so my message was being shared with a ton of people…at the very least I started a conversation, so it’s something, which is more than nothing.

Social media appear to be used as a platform to spread awareness, protest inequities, educate the public, engage others in critical dialogue and simply, be heard. Other participants, such as Peter (White, gay man), also acknowledged the importance of speaking out to confront social injustices in sport:

After I came out, I kind of became the go-to person for LGBTQ[2S] sport issues. My voice was almost one of the only ones out there for so long, so it was important for me to stand my ground, not just for me, but for other athletes who haven’t found the courage for expression…This is the last thing I would have wished for myself, but I felt responsible. It was where life went, and it was necessary in the time. And it still is necessary to talk about…at the time, the media and some teammates were so against me bringing my sexuality into sport, and I often said to them, ‘you don’t get a free pass. You don’t just get to dismiss this as a non-issue. Bullshit. It’s an issue.’

For Peter (White, gay man), speaking out on LGBTQ2S^3^ rights and issues was perceived as his duty to advocate for the inclusion of other gay athletes who may have feared disclosing their sexuality.

Participants expressed the importance of education to increase people’s awareness about the lived experiences of equity-deserving groups. For example, Paul (Black, heterosexual man) stated ‘it should be mandatory for coaches to take a course or seminar on Black history. Especially if you are a White coach with Black athletes. If they understand the struggle, they might do better’. Similarly, Danielle (White, physically disabled and non-binary queer) explained ‘One act a sport leader can learn to do is to introduce themselves with pronouns and actually use everyone’s pronouns and self-correct when they use improper pronouns. Pronoun education is a simple method of enhancing inclusion’. When discussing education, Danielle acknowledged the shortcomings of Canada’s coach education system around disability sport and advocated for improvements in this area:

I’d say the training for disabled folks is lacking. Clearly, we aren’t even doing basic disability 101 with our parasport coaches let alone anyone outside of that…I think education is a big part of it, but I’m also a big fan of practices…how do we get to a point where we tie education into a capacity for people to apply it to their own context? Not just going to a place and listening to a seminar and checking a box and eventually going home without the skills to say look at my own policies and practices, but giving people the practical skills to ask, ‘are my policies inclusive?’ ‘Do my hiring practices reflect a commitment to diversity?’

Finally, some participants explained how community engagement is critical to overcome the structural barriers embedded in sport. Ruth (White, heterosexual physically disabled woman) stated:

If Safe Sport included disabled people in conversations, planning, or even hiring processes, then maybe sport would look a little more accessible. We’d have wheelchair ramps or adapted equipment readily available in different spaces, we’d have posters or commercials starring successful Paralympic athletes. Maybe the Paralympics would be held before the Olympics, instead of just being an afterthought. The same can be said with other marginalized groups. They wouldn’t have to be accommodated for if they were included from the start. I’m probably biased but I think it’s harder for disabled people because a lot of times we can’t physically participate because a space or sport isn’t designed with us in mind.

Similarly, Mary (Middle Eastern, heterosexual woman) explained how engaging women in leadership and decision-making processes may facilitate a culture shift that better protects women in sport:

I think Safe Sport should be a catalyst for creating safer spaces for women and that can only happen if there are more women around. In a sport like swimming where we have a history of abuse and our uniforms are revealing enough, it just seems like we need more women in these spaces. It would be great if I can have discussion with women coaches about my body and my needs. It would be great if we were given similar recognition as the men. It’s not happening under the current leadership at [university] because it’s so male dominant. But if we empower women, whether as coaches or athletes or in any other role, then maybe we can change that.

## Discussion

The current study explored equity-deserving athletes’ perspectives and experiences of Safe Sport. Contrary to the abuse prevention perspectives of Safe Sport that tend to characterise the positions advanced by many sport organisations (Kerr et al., 2020), the current participants’ understandings of Safe Sport expanded beyond the prevention of physical, sexual or psychological abuse to the development of fair, inclusive, accessible and non-discriminatory sport cultures. According to the participants, Safe Sport is an improbable and idealistic concept that is not likely to be fully realised by these athletes because they are discriminated against for how they identify. All the participants described being discriminated against through verbal and behavioural microaggressions, as well as systemic barriers imposed by the sport environment and systems, thus influencing the perception of their sport experiences being unsafe. Participants claimed that Safe Sport was relevant and attainable for athletes who appear to fit within societal-normative identities (e.g., White, straight, able-bodied and man) more so than for athletes from equity-deserving groups (e.g., Black, gay, disabled and woman). The perception that Safe Sport is an inaccessible and unattainable standard of sport for these athletes may stem from their experiences of racism, sexism, ableism and homophobia in sport.

The social exclusion and discrimination of equity-deserving groups in sport have been described as an ongoing human rights violation that lacks the recognition and validation of daily experiences and situations of diverse populations (Donnelly and Coakley, 2002). The historic segregation of Black and White athletes (Stride et al., 2014), the exclusion of women (Tjønndal, 2019) and the persecution of transgender athletes (Love, 2017) represent some of the various ways in which the human rights of equity-deserving athletes have been infringed upon in sport (Donnelly, 2008; Teetzel, 2014) and have created physically and psychologically unsafe sport environments. The notion of social exclusion suggests the disadvantages experienced by equity-deserving groups result from various political structures and social processes (Labonte, 2004). Subsequently, contemporary moral claims of sport that assume fair play, universality and character development have not been, and continue not to be, inclusive of all participants, but rather have privileged colonial, White, upper-class, able-bodied and heterosexual men (Kidd and Donnelly, 2000). The findings from this study suggest similar forms of privilege continue to exist in sport in the form of structural barriers, consequently depriving equity-deserving athletes inclusive, accessible and Safe Sport opportunities.

Historically, sport has been described as ‘a laboratory for masculinity’ (Bonde, 2009, p. 1315), offered exclusively to specific types of men for the purposes of promoting physical and character development (Pike, 2015), along with masculine virtues of independence, discipline and courage (Bonde, 2009). The contemporary reproduction of historical narratives of hegemonic masculinity that classify men as aggressive, competitive, able-bodied and heterosexual (Messner, 1992; Anderson, 2005) may alienate those who do not assumingly fit that profile. Wellard (2002) acknowledges that sport participation is ‘dominated by a particular form of masculinity based on competitiveness, aggression and elements of traditional understandings of the sporting male…’ (p. 235). Moreover, Dashper (2012, p. 1111) explains how ‘sport’s role as a ‘maker of men’ has been facilitated by the exclusion of all things feminine and unmasculine, marginalizing, silencing and frequently excluding gay men’. Assumptions pertaining to masculinity have created unsafe experiences for gay and Black athletes, alike. Gay men athletes’ scepticism of Safe Sport may stem from experiences of verbal microaggressions (e.g., stereotyping), the reinforcement of hypermasculine values and widespread use of homophobic terms, which collectively reinforce a sport environment characterised by prejudice, intolerance and exclusion (Anderson, 2002, 2005; Dashper, 2012; Anderson et al., 2016; Lee et al., 2018; Baiocco et al., 2020), all of which contribute to perceptions of the sport environment as unsafe.

The embodiment of hegemonic perspectives of race, sexuality and masculinity in sport collectively represents structural forms of oppression that continue to perpetuate sport spaces deemed unsafe for equity-deserving athletes. For example, gay athletes are believed to be consumed by their aesthetics and are often assumed to be White and feminised by their participation in nonaggressive sports, such as swimming, gymnastics, cheerleading, running, ice skating and diving (Anderson and McCormack, 2010). Homophobic terms, such as ‘queer’, ‘pussy’ and ‘fag’, used to criticise athletes who do not conform to heteronormative masculine narratives (Dashper, 2012), were reportedly experienced by the two gay athletes who participated in this study. In comparison, assumptions exist that ‘Black men compete for faster times or harder hits…Black athletes are often portrayed as violent men in heterosexualized sporting spaces…Black athletes are thought to sweat, fuck, and fight…’ (Anderson and McCormack, 2010, p. 950). Claims of masculinity that assume Black athletes are naturally aggressive, strong and innately capable of jumping high and running fast (Bhana, 2008) have created an unsafe culture of sport characterised by the normalised use of racial microaggressions. Racial microaggressions may be perpetuated to reinforce a brand of Black masculinity associated with superior athletic abilities and thus, enhanced opportunities for winning and success. Consequently, Black male athletes who are incapable of expressing this brand of masculinity are subjected to experiencing behavioural microaggressions that exclude them from participation in sport, such as sitting on the bench for not playing as a male Black athlete is expected to play.

Individuals who identify with a disability must negotiate the relationship between social constructions of disability, the body and identity (Huang and Brittain, 2006). The athletes in this study who identified with a disability admitted to experiencing verbal, behavioural and environmental microaggressions from their coaches, teammates, and the overarching ableist design of high-performance sport. Consistent with the findings of DePauw and Gavron (2005), the participants suggested their experiences of discrimination stem from societal and sport-related assumptions that participants with a disability are incapable, frail and weak. The lived experiences of (un)Safe Sport endured by the participants with a disability reveal how medical discourses of disability remain, with a narrative of ‘cannot’ on athletes with disabilities (Huang and Brittain, 2006). Perceptions of inferiority perpetuate existing concerns surrounding the safeguarding of athletes with disabilities, which in the context of sport development initiatives, has been described as ‘marginal policy priority’ (Smith, 2015, p. 157), evidenced by a shortage of early sport experiences for athletes with disabilities, lack of resources, inadequate training opportunities and limited understanding of how to meaningfully include people with disabilities in sport (DePauw and Gavron, 2005).

The intersectional oppression endured by participants with disabilities who also were female or queer and non-binary reinforced narratives of weakness and inability, consequently heightening their experiences of exclusion and alienation (Remedios and Snyder, 2018). Sport has always been organised based upon binary categories of ability and gender, insofar that able-bodied and participants with disabilities or men and women compete in separate leagues and certain sports are characterised as being masculine or feminine (Davis, 2017). The separation between men and women in sport is heightened by hegemonic masculine norms, misogynistic perceptions of women in the media and the implementation of different rules in sports for men and women (e.g., women use a smaller basketball and there is no body checking in women’s hockey; Young and White, 2007; Flores et al., 2020). Sport’s binary organisation disregards the spectrum of identities with which one may identify. For example, only recently have we seen increased scholarly attention pertaining to the participation of transgender athletes (Anderson and Travers, 2017). The underrepresentation of genders beyond the binary reveals how gender-normative stereotypes function to exclude participants from reaping the benefits of accessible and inclusive Safe Sport.

### Recommended Changes to Advance Safe Sport

The unique perceptions and experiences of (un)Safe Sport reported by the participants exposes gaps in current Safe Sport interpretations and practices. Further, the perceived ineffectiveness of Safe Sport in protecting equity-deserving athletes may be a result of Safe Sport efforts that rely on broad, open-door practices^4^ to promote (or force) assimilation of equity-deserving athletes into a sport system that is inherently exclusionary, rather than fostering spaces for diverse participants to meaningfully enter and participate (Frisby and Ponic, 2013). To advance Safe Sport, policy and education must include the prevention of social injustices, and the promotion of inclusive, rights-based sport that accommodates the unique needs of diverse, equity-deserving participants. For example, sport organisations may consider mandating all sport stakeholders to complete anti-discriminatory and/or anti-colonial education. Additionally, sport organisations and different levels of government responsible for the governance of sport must integrate and enforce equity/inclusion policies that address consequences for discriminatory behaviours, requirements for equal employment opportunities and standards regarding the accessibility of resources and sport spaces (Joseph et al., 2021). Implementing education and policies related to social injustice issues, such as racism, homophobia, misogyny and ableism, within the Safe Sport discourse demonstrates a rejection of the ‘one size fits all’ mentality that assumes everyone understands and experiences Safe Sport similarly. Further, it empowers stakeholders of Safe Sport to consider how sport can be organised, developed and managed with principles of inclusion, equity, accessibility and acceptance at the forefront (Gurgis and Kerr, 2021). Additionally, sport organisations must commit to removing barriers (e.g., education, experience) that interfere with the recruitment of equity-deserving leaders (Joseph et al., 2021); prioritising diverse leadership across Safe Sport may further shape how Safe Sport is conceptualised and advanced. As well, consistent with the findings, the advancement of Safe Sport for underrepresented groups is contingent on having diverse leaders who can empathise with equity-deserving athletes. Finally, it is essential that sport organisations enhance accountability measures (Joseph et al., 2021) to confront the complacent nature of Safe Sport, which to this point, has failed to consider the subtle, yet harmful ways in which sport and its participating stakeholders affect equity-deserving athletes. Accountability measures may include publicly committing to instilling changes that enhance the representation and experiences of equity-deserving groups (Joseph et al., 2021), ongoing collection of demographic data, evaluation data including athletes’ experiences and the linking of funding with the achievement of change.

### Limitations and Future Directions

The study was limited by having a small sample of participants representing various equity-deserving groups (e.g., only two Black athletes, two gay athletes and one non-binary athlete) Additionally, not every equity-deserving group was reflected in this study (e.g., transgender and Indigenous). Finally, the study was limited to the perspectives of high-performance athletes in Canada. Future Safe Sport research must consider the perspectives of multiple stakeholders (e.g., equity-deserving administrators, coaches and/or researchers). Further, exploring the perspectives and experiences of youth athletes, athletes outside of a high-performance context, and international equity-deserving groups, may also yield novel interpretations of Safe Sport or introduce new experiences of Safe Sport endured by equity-deserving participants. Additionally, the use of diverse methods of inquiry, such as participant observation, may facilitate new learnings about how equity-deserving stakeholders experience Safe Sport. Finally, intervention research should explore how Safe Sport strategies can be enhanced to better safeguard equity-deserving athletes from experiences of violence in sport. This, in turn, may lead sport organisations to adopting and advancing more all-encompassing interpretations of Safe Sport grounded in principles of fairness, accessibility, inclusion and equal rights.

## Conclusion

This study explored the equity-deserving athletes’ perspectives, experiences of and recommendations for advancing Safe Sport. The findings indicated that participants’ perspectives and experiences of Safe Sport differed from those of White, male, heterosexual and able-bodied athletes, and that Safe Sport initiatives, which focus on abuse prevention, inadequately recognise or address discrimination and microaggressions as forms of violence. The participants recommended that Safe Sport should extend beyond the common understanding of abuse prevention to include anti-discrimination, as each athlete reported experiences of verbal, behavioural and structural forms of harm, which ultimately shaped their understanding of Safe Sport. As such, according to the athletes, topics, such as racism, ableism, sexism, misogyny and heterosexism, are critical to address within the Safe Sport movement, as these experiences uniquely define the (un)safe experiences of marginalised athletes in sport. Overall, although sport has progressed significantly since its inception, especially *via* Safe Sport, the prejudicial treatment of equity-deserving groups in sport continues to be characterised by epistemic injustice (Pratt, 2019).

## Data Availability Statement

The datasets presented in this article are not readily available because the datasets are not anonymized. Requests to access the datasets should be directed to the corresponding author: joseph_gurgis@cbu.ca.

## Ethics Statement

The studies involving human participants were reviewed and approved by University of Toronto Health Sciences Research Ethics Board. The participants provided their written informed consent to participate in this study.

## Author Contributions

JJG contributed to the conceptualization of the study, collected and analysed the data, wrote each section, and edited the manuscript. GK contributed to the conceptualization of the study and the analysis and writing, and thoroughly edited the paper. SD thoroughly edited the paper and offered conceptual recommendations. All authors contributed to the article and approved the submitted version.

## Conflict of Interest

The authors declare that the research was conducted in the absence of any commercial or financial relationships that could be construed as a potential conflict of interest.

## Publisher’s Note

All claims expressed in this article are solely those of the authors and do not necessarily represent those of their affiliated organizations, or those of the publisher, the editors and the reviewers. Any product that may be evaluated in this article, or claim that may be made by its manufacturer, is not guaranteed or endorsed by the publisher.

## Footnotes

^1^For the purpose of this paper, Safe Sport refers to a philosophy, whereas SafeSport refers to the entity U.S. Center for SafeSport. As a philosophy, there are varying, inconsistent definitions of what Safe Sport entails (Kerr et al., 2020).

^2^Universities have used the term equity-seeking to acknowledge the underrepresentation of particular groups, such as Indigenous Peoples, women and individuals with disabilities; the term equity-deserving has emerged in place of equity-seeking, which ‘shifts attention towards Black, Indigenous, People of Colour, Women, People with (dis)abilities, and LGBTQ[2S] people as deserving of representation and belonging’ (Global Water Futures, 2021, p. 7). Additionally, equity-deserving challenges the assumption that under-represented groups need to seek equity, given their fundamental right to experience it (Garbutt, 2020).

^3^Lesbian, Gay, Bisexual, Transexual, Queer and Two-Spirit (LGBTQ2S) is the preferred acronym, as it is inclusive of the ways in which Indigenous people describe their gender, sexual and spiritual identities (Filice, 2020).

^4^‘Sport for all policies’ (Frisby and Ponic, 2013, p. 389) remain ineffectual due to the current, exclusionary structure of sport (Donnelly and Coakley, 2002; Collins, 2014). This is especially true in high-performance sport, where ideological conformity, normalisation of harmful practices, nationalism, abuse and over-emphasis on winning are prevalent and contribute to unsafe experiences, especially for equity-deserving participants (Collins, 2014).

## References

[ref1] Alase A. (2017). The interpretative phenomenological analysis (ipa): a guide to a good qualitative research approach. Int. J. Educ. Lit. Stud. 5, 9–19. doi: 10.7575/aiac.ijels.v.5n.2p.9

[ref2] Alexander K. Stafford A. Lewis R. (2011). The Experiences of Children Participating in Organized Sport in the UK. London: NSPCC.

[ref3] Anderson E. (2002). Openly gay athletes: contesting hegemonic masculinity in a homophobic environment. Gend. Soc. 16, 860–877. doi: 10.1177/089124302237892

[ref4] Anderson E. (2005). Orthodox and inclusive masculinity: competing masculinities among heterosexual men in a feminized terrain. Sociol. Perspect. 48, 337–355. doi: 10.1525/sop.2005.48.3.337

[ref5] Anderson E. Magrath R. Bullingham R. (2016). Out in Sport: The Experiences of Openly Gay and Lesbian Athletes in Competitive Sport. London: Routledge.

[ref6] Anderson E. McCormack M. (2010). Intersectionality, critical race theory, and American sporting oppression: examining black and gay male athletes. J. Homosex. 57, 949–967. doi: 10.1080/00918369.2010.503502, PMID: 20818524

[ref7] Anderson E. Travers A. (2017). Transgender Athletes in Competitive Sport. London: Routledge.

[ref8] Baiocco R. Pistella J. Salvati M. Ioverno S. Lucidi F. (2020). Sexual prejudice in sport scale: a new measure. J. Homosex. 67, 489–512. doi: 10.1080/00918369.2018.1547560, PMID: 30507299

[ref9] Berry K. Kowalski K. C. Ferguson L. J. McHugh T. F. (2010). An empirical phenomenology of young adult women exercisers’ body self-compassion. Qual. Res. Sport Exerc. 2, 293–312. doi: 10.1080/19398441.2010.517035

[ref10] Bhana D. (2008). ‘Six packs and big muscles, and stuff like that’. Primary school-aged south African boys, black and white, on sport. Br. J. Sociol. Educ. 29, 3–14. doi: 10.1080/01425690701728654

[ref11] Biggerstaff D. Thompson A. R. (2008). Interpretative phenomenological analysis (IPA): a qualitative methodology of choice in healthcare research. Qual. Res. Psychol. 5, 214–224. doi: 10.1080/14780880802314304, PMID: 30949242

[ref12] Bonde H. (2009). The time and speed ideology: 19th century industrialisation and sport. Int. J. Hist. Sport 26, 1315–1334. doi: 10.1080/09523360903057419

[ref13] Boocock S. (2002). The child protection in sport unit. J. Sex. Aggress. 8, 99–106. doi: 10.1080/13552600208413342, PMID: 34828732

[ref14] Burdsey D. (2011). That joke isn’t funny anymore: racial microaggressions, color-blind ideology and the mitigation of racism in English men’s first-class cricket. Sociol. Sport J. 28, 261–283. doi: 10.1123/ssj.28.3.261

[ref15] Canadian Institutes of Health Research (2018). Natural Sciences and Engineering Research Council of Canada, and Social Sciences and Humanities Research Council of Canada, Tri-Council Policy Statement: Ethical Conduct for Research Involving Humans. Available at: https://ethics.gc.ca/eng/documents/tcps2-2018-en-interactive-final.pdf (accessed December, 2018).

[ref16] Coaching Association of Canada (2020). Safe Sport training. Available at: https://safesport.coach.ca/ (accessed November 24, 2021).

[ref17] Collins M. (2014). Sport and Social Exclusion. London: Routledge.

[ref19] Creswell J. W. Plano Clark V. L. (2011). Designing and Conducting Mixed Methods Research. New York: Sage Publications.

[ref20] Dashper K. (2012). ‘Dressage is full of queens!’ masculinity, sexuality and equestrian sport. Sociology 46, 1109–1124. doi: 10.1177/0038038512437898

[ref21] Davis H. F. (2017). Beyond Trans: Does Gender Matter? New York: New York University Press.

[ref22] Dean C. Rowan D. (2014). The social worker’s role in serving vulnerable athletes. J. Soc. Work. Pract. 28, 219–227. doi: 10.1080/02650533.2013.817987

[ref23] Department of Education. (2018). Working together to safeguard children: a guide to inter-agency working to safeguard and promote the welfare of children. Available at: https://assets.publishing.service.gov.uk/government/uploads/system/uploads/attachment_data/file/942454/Working_together_to_safeguard_children_inter_agency_guidance.pdf (accessed July, 2018).

[ref25] DePauw K. P. Gavron S. J. (2005). Disability Sport. Champaign, IL: Human Kinetics.

[ref26] Donnelly P. (2008). Sport and human rights. Sport Soc. 11, 381–394. doi: 10.1080/17430430802019326, PMID: 35088498

[ref27] Donnelly P. Coakley J. J. (2002). The Role of Recreation in Promoting Social Inclusion. Toronto: Laidlaw Foundation.

[ref28] Filice M. (2020). Two-spirit. The Canadian Encyclopedia. Available at: https://www.thecanadianencyclopedia.ca/en/article/two-spirit (accessed July 3, 2020)

[ref29] Flores A. R. Haider-Markel D. P. Lewis D. C. Miller P. R. Tadlock B. L. Taylor J. K. (2020). Public attitudes about transgender participation in sports: the roles of gender, gender identity conformity, and sports fandom. Sex Roles 83, 382–398. doi: 10.1007/s11199-019-01114-z

[ref30] Frisby W. Ponic P. (2013). “Sport and social inclusion,” in Sport Policy in Canada. eds. Thibault L. Harvey J. (Ottawa, ON: University of Ottawa Press), 381–403.

[ref31] Garbutt C. (2020). Talk Into Action: What’s Next for the National Dialogues. Scarborough: University of Toronto Scarborough.

[ref32] Global Water Futures. (2021). Equity, Diversity, & Inclusion: Global Water Futures: 2021–2023 Draft Strategy for Consultation. Saskatoon: Global Water Futures.

[ref34] Gurgis J. J. Kerr G. (2021). Sport administrators’ perspectives on advancing safe sport. Front. Sports Act. Living 3: 630071. doi: 10.3389/fspor.2021.630071, PMID: 34169275 PMC8217753

[ref35] Hayhurst L. Kay T. Chawansky M. (2016). Beyond Sport for Development and Peace: Transnational Perspectives on Theory and Practice. London: Routledge.

[ref36] Huang C. J. Brittain I. (2006). Negotiating identities through disability sport. Sociol. Sport J. 23, 352–375. doi: 10.1123/ssj.23.4.352

[ref37] International Olympic Committee. (1997). Olympic Charter. Lausanne: IOC Sport for All Commission.

[ref38] Jones S. Torres V. Arminio J. (2014). Negotiating the Complexities of Qualitative Research in Higher Education. London: Routledge.

[ref39] Joseph J. Razack S. McKenzie B. (2021). Are we One? The Ontario University Athletics Anti-Racism Report. IDEAS Research Lab, University of Toronto.

[ref40] Kaskan E. R. Ho I. K. (2014). Microaggressions and female athletes. Sex Roles 74, 275–287. doi: 10.1007/s11199-014-0425-1

[ref41] Kelly E. M. (2013). The Jerry Sandusky effect: child abuse reporting laws should no longer be “don't ask, don’t tell”. Univ. Pittsbg. Law Rev. 75, 209–233. doi: 10.5195/lawreview.2013.305

[ref42] Kennedy S. Grainger J. (2006). Why I didn’t Say Anything: The Sheldon Kennedy Story. London, ON: Insomniac Press.

[ref43] Kerr G. Kidd B. Donnelly P. (2020). One step forward, two steps back: the struggle for child protection in Canadian sport. Soc. Sci. 9, 68–83. doi: 10.3390/socsci9050068

[ref44] Kidd B. Donnelly P. (2000). Human rights in sports. Int. Rev. Sociol. Sport 35, 131–148. doi: 10.1177/101269000035002001

[ref46] Labonte R. (2004). Social inclusion/exclusion: dancing the dialectic. Health Promot. Int. 19, 115–121. doi: 10.1093/heapro/dah112, PMID: 14976179

[ref47] Larkin M. Thompson A. R. (2012). “Interpretative phenomenological analysis in mental health and psychotherapy research,” in Qualitative Research Methods in Mental Health and Psychotherapy: A Guide for Student and Practitioners. eds. Harper D. Thompson A. R. (Chichester, Ltd: John Wiley & Sons), 101–116.

[ref48] Lee S. Bernstein M. B. Etzel E. F. Gearity B. T. Kuklick C. R. (2018). Student-athletes’ experiences with racial microaggressions in sport: a Foucauldian discourse analysis. Qual. Rep. 23, 1016–1043. doi: 10.46743/2160-3715/2018.3095

[ref49] Love A. (2017). “The tenuous inclusion of transgender athletes in sport,” in Transgender Athletes in Competitive Sport. eds. Anderson E. Travers A. (London: Routledge), 194–205.

[ref50] Masters J. Veselinovic M. (2018). Barry Bennell: 31 years in jail for soccer’s ‘devil incarnate’. CNN. Available at: https://www.cnn.com/2018/02/19/sport/barry-bennell-sexual-abuse-sentenced-intl/index.html (accessed February 20, 2018)

[ref51] Messner M. A. (1992). Power at Play: Sports and the Problem of Masculinity. Boston, MA: Beacon Press.

[ref52] Mountjoy M. Brackenridge C. Arrington M. Blauwet C. Carska-Sheppard A. Fasting K.. (2016). International Olympic Committee consensus statement: harassment and abuse (non-accidental violence) in sport. Br. J. Sports Med. 50, 1019–1029. doi: 10.1136/bjsports-2016-096121, PMID: 27118273

[ref53] Moustakas C. (1994). Phenomenological Research Methods. Thousand Oaks: Sage.

[ref54] Parent S. Vaillancourt-Morel M. (2020). Magnitude and risk factors for interpersonal violence experienced by Canadian teenagers in the sport context. J. Sport Soc. Issues 45, 528–544. doi: 10.1177/0193723520973571

[ref55] Parson L. (2019). “Considering positionality: the ethics of conducting research with marginalized groups,” in Research Methods for Social Justice and Equity in Education. eds. Strunk K. K. Locke L. A. (London: Palgrave Macmillan), 15–32.

[ref56] Patton M. Q. (2002). Qualitative Research and Evaluation Methods. 3rd Edn. Thousand Oaks, CA: Sage Publications.

[ref57] Pike E. C. J. (2015). Assessing the sociology of sport: on age and ability. Int. Rev. Sociol. Sport 50, 570–574. doi: 10.1177/1012690214550009

[ref58] Pratt B. (2019). Inclusion of marginalized groups and communities in global health research priority-setting. J. Empir. Res. Hum. Res. Ethics 14, 169–181. doi: 10.1177/1556264619833858, PMID: 30866721

[ref59] Remedios J. D. Snyder S. H. (2018). Intersectional oppression: multiple stigmatized identities and perceptions of invisibility, discrimination, and stereotyping. J. Soc. Issues 74, 265–281. doi: 10.1111/josi.12268

[ref60] Simien E. M. Arinze N. McGarry J. (2019). A portrait of marginality in sport and education: toward a theory of intersectionality and race-gendered experiences for black female college athletes. J. Women Polit. Policy 40, 409–427. doi: 10.1080/1554477X.2019.1614865

[ref61] Smith J. A. (2004). Reflecting on the development of interpretative phenomenological analysis and its contribution to qualitative research in psychology. Qual. Res. Psychol. 1, 39–54. doi: 10.1191/1478088704qp004oa

[ref78] Smith J. A. Osborn M. (2008). Interpretative phenomenological analysis. In Smith J. A. (Ed.). Qualitative psychology: A practical guide to research methods 53–80. Sage Publications.

[ref62] Smith A. (2015). “Safeguarding the welfare of disabled people in sport: some policy issues and considerations,” in Safeguarding, Child Protection and Abuse in Sport: International Perspectives in Research, Policy and Practice. eds. Lang M. Hartill M. (New York: Routledge), 153–162.

[ref63] Smith J. A. Flowers P. Larkin M. (2009). Interpretative Phenomenological Analysis: Theory, Method, and Research. Thousand Oaks, CA: Sage

[ref64] Sparkes A. C. Smith B. (2013). Qualitative Research Methods in Sport, Exercise and Health: From Process to Product. London: Routledge.

[ref65] Stride C. Thomas F. Smith M. M. (2014). Ballplayer or barrier breaker? Branding through the seven statues of Jackie Robinson. Int. J. Hist. Sport 31, 2164–2196. doi: 10.1080/09523367.2014.923840, PMID: 32201726

[ref66] Strunk K. K. Locke L. A. (2019). Research Methods for Social Justice and Equity in Education. London: Palgrave Macmillan.

[ref67] Sue D. W. Capodilupo C. M. Torino G. C. Bucceri J. M. Holder A. M. B. Nadal K. L.. (2007). Racial microaggressions in everyday life: implications for clinical practice. Am. Psychol. 62, 271–286. doi: 10.1037/0003-066X.62.4.271, PMID: 17516773

[ref68] Tamminen K. A. Holt N. L. Neely K. C. (2013). Exploring adversity and the potential for growth among elite female athletes. Psychol. Sport Exerc. 14, 28–36. doi: 10.1016/j.psychsport.2012.07.002

[ref69] Teetzel S. (2014). The onus of inclusivity: sport policies and the enforcement of the women’s category in sport. J. Philos. Sport 41, 113–127. doi: 10.1080/00948705.2013.858394

[ref70] Tjønndal A. (2019). I don’t think they realise how good we are’: innovation, inclusion and exclusion in women’s Olympic boxing. Int. Rev. Sociol. Sport 54, 131–150. doi: 10.1177/1012690217715642

[ref71] U.S. Center for SafeSport. (2021). 2020 athlete culture and climate survey. Available at: https://uscenterforsafesport.org/wp-content/uploads/2021/07/CultureClimateSurvey_ExternalReport_071421_Final.pdf (accessed July 14, 2021).

[ref72] van Manen M. (1990). Researching Lived Experience: Human Science for an Action Sensitive Pedagogy. Albany, NY: SUNY Press.

[ref73] Vertommen T. Schipper-van Veldhoven N. Wouters K. Kampen J. K. Brackenridge C. H. Rhind D. J. A.. (2016). Interpersonal violence against children in sport in the Netherlands and Belgium. Child Abuse Negl. 51, 223–236. doi: 10.1016/j.chiabu.2015.10.006, PMID: 26516053

[ref74] Vogt W. P. (2005). Dictionary of Statistics and Methodology: A Nontechnical Guide for the Social Sciences. Los Angeles, CA: Sage.

[ref75] Wellard I. (2002). Men, sport, body performance and the maintenance of ‘exclusive masculinity’. Leis. Stud. 21, 235–247. doi: 10.1080/0261436022000030641

[ref76] Willson E. Kerr G. Stirling A. Buono S. (2021). Prevalence of maltreatment among Canadian national team athletes. J. Interpers. Violence. 36, 1–23. doi: 10.1177/0886260521104509 PMC9554369 34549664

[ref77] Young K. White P. (2007). Sport and Gender in Canada. 2nd *Edn*. Don Mills, ON: Oxford.

